# From Trendelenburg to PERTs: Evolution in the Management of Massive Pulmonary Embolism

**DOI:** 10.14797/mdcvj.1345

**Published:** 2024-05-16

**Authors:** Pavan Thangudu

**Affiliations:** 1 Pulmonary Disease & Critical Care, Memorial Hermann Health System, The Woodlands, Texas, US

**Keywords:** massive pulmonary embolism, pulmonary embolism classification, pulmonary embolism misdiagnosis, pulmonary embolism response teams (PERTS)

## Abstract

Massive pulmonary embolism (MPE) is a serious condition affecting the pulmonary arteries and is difficult to diagnose, triage, and treat. The American College of Chest Physicians (AHA) and the European Society of Cardiology (ESC) have different classification approaches for PE, with the AHA defining three subtypes and the ESC four. Misdiagnosis is common, leading to delayed or inadequate treatment. The incidence of PE-related death rates has been increasing over the years, and mortality rates vary depending on the subtype of PE, with MPE having the highest mortality rate. The current definition of MPE originated from early surgical embolectomy cases and discussions among experts. However, this definition fails to capture patients at the point of maximal benefit because it is based on late findings of MPE. Pulmonary Embolism Response Teams (PERTs) have emerged as a fundamental shift in the management of MPE, with a focus on high-risk and MPE cases and a goal of rapidly connecting patients with appropriate therapies based on up-to-date evidence. This review highlights the challenges in diagnosing and managing MPE and emphasizes the importance of PERTs and risk stratification scores in improving outcomes for patients with PE.

## Introduction

Massive pulmonary embolism (MPE) remains one of the most difficult to diagnose, triage, and treat within the domain of PEs. There are two main classification approaches to PE, delineated geographically by the American Heart Association (AHA) representing the United States (US) and the European Society of Cardiology (ESC) representing Europe (Tables 1, 2).

**Table 1:** American Heart Association classification of pulmonary embolism. BP: blood pressure; BPM: beats per minute; RV: right ventricular; BNP: brain natriuretic peptide; EKG: electrocardiogram; CT: computer tomography


**Massive pulmonary embolism**	Sustained hypotension (systolic BP < 90 mm Hg for 15 min or requiring ionotropic support), ***or*** pulselessness, ***or*** sustained heart rate < 40 BPM with signs/symptoms of shock

**Sub-massive pulmonary embolism**	Systolic BP > 90 mm Hg and RV dysfunction or myocardial necrosis defined by: RV dilation (apical 4-chamber RV diameter divided by LV diameter > 0.9), or RV systolic dysfunction on echocardiography, ***or*** RV dilation (4-chamber RV diameter divided by LV diameter > 0.9) on CT, ***or*** elevation of BNP (> 90 pg/mL), or N-terminal pro-BNP (> 500 pg/mL), ***or*** EKG changes (new complete or incomplete right bundle-branch block, anteroseptal ST elevation or depression, or anteroseptal T-wave inversion), ***or*** elevation of troponin I (> 0.4 ng/mL) or troponin T (> 0.1 ng/mL)

**Low-risk pulmonary embolism**	Absence of adverse prognosis markers defining massive or submassive PE


**Table 2:** European Society of Cardiology Classification of High-Risk Pulmonary Embolism. BP: blood pressure; CTPE: computed tomography pulmonary angiography; PESI: Pulmonary Embolism Severity Index; sPESI: simplified PESI; TTE: transthoracic echocardiogram; PE: pulmonary embolism; RV: right ventricular; CTPA: computed tomography pulmonary angiography


**High risk**	Cardiac arrest, obstructive shock (systolic BP < 90 mm Hg despite an adequate filling status, in combination with end-organ hypoperfusion), or persistent hypotension (systolic BP drop > 40 mm Hg for > 15 min, not caused by new-onset arrhythmia, hypovolemia, or sepsis), or CTPA with hemodynamic instability and clinical PESI score of Class III-V or sPESI of > 1

**Intermediate high risk**	Signs of RV dysfunction on TTE (or CTPA) with elevated cardiac biomarker levels

**Intermediate low risk**	Clinical parameters of PE severity and/ or comorbidity: PESI class III–V or sPESI ≥ 1 with the presence of either RV dysfunction on TTE or CTPA. Cardiac biomarkers may be normal or elevated

**Low risk**	No cardiac markers, no RV dysfunction, no hemodynamic instability


The AHA describes three subtypes of PE: simple, sub-massive, and massive. A simple PE is defined as a thrombus that has migrated from the body’s deep veins to the pulmonary circulation without evidence of right cardiac dysfunction. Sub-massive pulmonary embolism (SMPE) is defined by the presence of a PE and evidence of right ventricular (RV) cardiac dysfunction. AHA guidelines define MPE as the presence of PE, systolic blood pressure (SBP) < 90 mm Hg or a transient drop in SBP of > 40 mm Hg lasting longer than 15 minutes, and not caused by sepsis, new-onset arrhythmia, or hypovolemia.^1,2^

The ESC 19 guidelines describe four different subtypes of PE: low-risk, intermediate low-risk, intermediate high-risk, and MPE. The guidelines have broadened the definition of MPE to include a PE that is causing cardiac arrest, or the presence of obstructive shock defined by SBP < 90 mm Hg or vasopressors required to achieve SBP > 90 mm Hg despite adequate filling status and the presence of end-organ hypoperfusion while also including the AHA guideline definition criteria.^3^

Misdiagnosis of patients with PE is common, with approximately 30% of emergency department patients initially diagnosed with an alternative condition. Inpatients have a 50% risk of being misdiagnosed, and approximately 40% of intensive care unit (ICU) patients are found on autopsy to have a PE that was not diagnosed before death.^4^

The estimated incidence of all PE in the US per the Centers for Disease Control (CDC) is approximately 900,000 cases per year.^5^ The incidence of PE and death rates have been increasing over the last 20 years.^6,7^ Due to difficulties in diagnosing PE, the reported number of cases is likely lower than the actual total. The incidence of MPE remains unknown. The mortality of PE increases proportionally with the pooled organ injury from PE, with estimated mortality being 5% to 15% for simple PE, 15% to 25% for SMPE, and 30% to 60% for MPE.^8^

Acute MPE results from significant obstruction of the main pulmonary arteries resulting in increased afterload on the pulmonary artery, which leads to decreased RV cardiac output. The resultant drop in RV cardiac output leads to lower left ventricular (LV) preload and a subsequent decrease in cardiac output of the left ventricle. This causes arterial hypotension, leading to decreased coronary blood flow and, in turn, a reduction in RV contractility. This results in dilatation of and increased wall tension on the right ventricle, which leads to a further reduction in cardiac output of the RV and ultimately more systemic hypotension. Acute MPE results from obstructive shock of the pulmonary arteries and cardiogenic shock from RV failure causing decreased systemic cardiac output from the left ventricle.^3,9^

Once the diagnosis of PE is made—usually based on a combination of history, physical examination, imaging, laboratory data, and risk stratification scores—triage of the disease into the three PE subtypes becomes the critical next steps as this is one of the most important clinical assessments made in determining the outcome of a patient with PE. Patient mortality is not solely predicted by subtypes of PE but also by clinical and biochemical markers assessing the harm of PE to the body. The presence of a heart rate greater than 100 bpm leads to a 4-fold increase in the risk of PE-related death compared to patients with PE and a heart rate less than 100 bpm.^8^ The presence of a shock index (heart rate/systolic blood pressure) greater than 0.89 in intermediate-risk PE predicts an 11.8-fold higher risk for in-hospital death than in patients with a shock index of less than 0.89.^10^ Venous lactate elevations have been shown to predict mortality and adverse outcomes in normotensive patients with PE.^11,12^

The FLASH PE registry revealed that 34.1% of patients with high-risk SMPE had a cardiac index of < 2.2 L/min^2^, confirming the presence of cardiogenic shock. However, these patients were misdiagnosed as not having shock when they actually had cardiac output failure, confirming MPE.^13^ Assessment and triage strategies should factor in cardiac output and failure of cardiac output, as well as SBP-based definitions, to guide clinical therapy for PE subtypes.

## Rethinking MPE

Understanding the failure of MPE’s current definition to capture patients at the point of maximal benefit requires reflection on the origin of the current definition. The first case of surgical pulmonary embolectomy was reported in 1908 by Dr. Friedrich Trendelenburg. This approach did not have a successful outcome until 1922. In 1935, Dr. Paul D. White published the first case series of four patients with acute cor pulmonale, which is thought to be the origin of the current definition of MPE. He reported in *Annals of Internal Medicine* that 50% of his patients died within 30 minutes, 70% within an hour, and more than 85% within 6 hours.^14,15^ Due to the high mortality rate and time constraints, cardiopulmonary bypass became a viable option for treating patients with MPE.

The first use of a cardiopulmonary bypass machine was reported in 1955. However, due to the high mortality and complications, the machines were used only for patients who failed to respond to medical management. It was not until 1961, when Dr. Denton Cooley performed a successful pulmonary embolectomy for acute MPE using a cardiopulmonary bypass machine, that their use for treating MPE gained more recognition.^16^ The next year, remarkable outcomes for MPE were reported when 13 of 19 moribund patients survived after receiving a surgical pulmonary embolectomy supported by cardiopulmonary bypass.^17^

During this same time, it became clear that a more uniform approach for diagnosing and treating PE was needed. The American College of Chest Physicians (CHEST) conference in 1962 featured a round table of experts exploring how to define MPE and served as a basis for the first guideline definition of MPE. In this discussion, Dr. John J. Sampson noted that a Bezold-Jarisch reflex—leading to hypotension that persisted beyond 30 minutes and was detected in animal models—was seen in the clinical syndrome of MPE and contributed to the hypotension from MPE.^18^ Reference was made to the Bezold-Jarisch reflex in animal models, and subsequently the AHA guidelines incorporated a drop in SBP of over 40 mm Hg for 15 minutes into the definition of MPE. However, the Bezold-Jarisch reflex is a late finding of MPE, whereas the diagnosis of MPE must be made as early as possible to benefit a patient’s survival. The current definitions of MPE fail to provide clinicians with the tools to safely diagnose a patient and offer the benefits of appropriate intervention.

## Pulmonary Embolism Response Teams

Perhaps the most fundamental shift in the trajectory of patients with MPE has been the introduction of Pulmonary Embolism Response Teams (PERTs), which coordinate care for high-risk and MPE patients. A systematic review and meta-analysis of PERT outcomes revealed that, compared to control groups, PERTs use more advanced interventions and show reduced complications and improved mortality across all cases of PE.^18^ While each PERT has its unique approach to treating PE, the standard across all PERTS is to focus on building pathways for patients diagnosed with PE to be rapidly matched with the most appropriate therapy based on current evidence.^19^ The first PERT was launched in 2012 at Massachusetts General Hospital and has since grown to over 100 teams across the world. This increased focus on PE through PERTs has led to earlier treatment, less bleeding, shorter hospital length of stay, and a trend towards lower mortality across all PE patients.

Aside from facilitating the rapid connection of patients with PE to beneficial therapies, PERTs have redefined the approach to risk stratification. The current risk tools predict mortality at 30 days but do not reliably predict short-term mortality during a hospital admission. Therefore, multiple clinical decision scores have been developed to further risk-stratify high-risk PE from low-risk PE. High-risk PE indicates a much higher likelihood of inpatient death. The most widely used scores include the Pulmonary Severity Index Score (PESI) and simplified PESI (sPESI), the BOVA score, the TELOS (thromboembolism lactate outcome study) score, the SHIELD (shock index, hypoxemia, lactate, cardiovascular dysfunction) score, and venous lactate.

Of these scoring systems, the PESI and sPESI scores have undergone the most validation thus far and therefore are used to rule out low-risk patients who do not require intervention; however, the PESI and sPESI are less reliable at differentiating acute mortality from 30-day mortality.^3,20^

The BOVA score, named after the study author, utilizes a weighted scoring system of HR, SBP, assessment of RV dysfunction, and SBP parameters to classify the risk of death into three subtypes: Stage I, a 4.4% risk of death; Stage II, an 18% risk of death; and Stage III, a 42% risk of death within 30 days.^20,21,22^ RV dysfunction was defined as the presence on echocardiography of RV/LV ratio > 0.9, sPAP > 30 mm Hg, RV dilatation, or RV free wall hypokinesis. The primary composite outcome of the score was PE-related death, recurrent PE, or hemodynamic collapse. The addition of lactate to the BOVA score was used to develop a 7-day mortality prediction score.

The TELOS score, which uses RVD, lactic acid, and troponin elevation, was prospectively developed and then further validated in another prospective trial in normotensive PE patients, which predicted adverse events at 7 days.^20^ The SHIELD score (shock index > 1, hypoxia, lactate elevation, and signs of RV dysfunction) was derived from a retrospective cohort study and then externally validated to predict 30-day mortality.^20^

Venous lactate was shown to be a predictor of mortality in PE patients independent of cardiac output state, troponin elevation, hypotension, or RVD.^11,12,20^ The addition of venous lactate to the ESC, BOVA, and TELOS scores was demonstrated to improve the prediction of early adverse events.^20^ However, the optimal plasma lactate cutoff level remains to be determined, and further studies are needed to refine the optimal cutoff criteria for short-term mortality.

Once MPE has been identified and stratified from low-risk or simple PE, it is imperative to quickly commence with treatment. Since MPE comprises two components, obstructive and cardiogenic shock, initial actions require resuscitation and stabilization of the patient as well as selecting the appropriate therapeutic strategy to optimize outcomes. The therapeutics for all MPE patients comprise a combination of anticoagulation, thrombolysis, and surgical pulmonary embolectomy. Based on ESC guidelines, consideration should be given for advanced therapies, including catheter-directed therapies such as mechanical circulatory support with venoarterial extracorporeal membrane oxygenation (VA-ECMO).^3^

Stabilization of cardiogenic shock requires judicious use of fluid. Since fluid has the ability to further worsen RV preload and cause worsening cardiac output, it should be restricted to patients who have a low central venous pressure.^3^ Diuretics also have been shown to decrease RV preload and improve the shock index in SMPE, with a trend toward lower mortality in the diuretic group compared to the volume expansion group.

The same study also may show the relative harm of volume expansion, more so than the benefits of diuresis.^23,24^ Furthermore, inodilators and inoconstrictors also may play a role. No one inodilator or inoconstrictor is advantageous in hemodynamically unstable PE. The trend is to select agents that minimize increases in pulmonary vasoconstriction while also balancing the negative impact of inodilators on systemic hypotension.^25^ The primary inoconstrictor most often recommended for this purpose is norepinephrine; the primary inodilators used are dobutamine and milrinone. Pulmonary vasodilators such as inhaled nitric oxide also have been used as a bridge to definitive therapy while stabilizing a patient with acute MPE and elevated RV afterload. While there is no evidence to support mortality reduction, it can be used to augment RV cardiac output.^26,27^

Simple anticoagulation remains the foundation for treating all PE, including MPE. Heparin is typically the preferred agent of choice, at a dose of 80 units/kg as an initial bolus followed by a fixed dose at 18 units/kg/hr titrated to rapidly achieve a therapeutic partial thromboplastin time. Combined with the use of thrombolytics for bleeding risk in appropriately screened patients, this offers some potential benefit of resolving the obstructive shock component of MPE.^28^

The use of thrombolysis in MPE is based on a randomized controlled trial of eight patients.^29^ Four patients received streptokinase and heparin and the remaining four were treated with heparin alone. All four patients who received heparin expired. This remains the only randomized controlled trial to date to study patients with MPE. The MOPET (moderate pulmonary embolism treated with thrombolysis) and MAPPET (moderate pulmonary embolism treated with thrombolysis) trials both excluded patients with MPE while studying the impact and dose of thrombolysis on patients with SMPE.^29,30,31^

Studies have shown that administering thrombolysis earlier can reduce the risk of complications. A retrospective study found that the complication rate from thrombolysis was lower when it was administered within 8.5 hours of admission to an emergency department.^15^ Even when treated with thrombolysis, the expected mortality from acute MPE with thrombolysis still ranges from 25% to 65%.

The International Cooperative Pulmonary Embolism Registry (ICOPER) noted underutilization of thrombolytics for MPE, with approximately 70% of patients with MPE not receiving thrombolysis. Compared with the subset of MPE patients who received thrombolysis, it showed no improvement in survival with thrombolysis. This suggests one reason why PERT is so successful in the triage and optimization of acute PE, as PERTs work towards reducing time to treatment initiation.

Despite an increasing trend toward the utilization of catheter-directed therapies for PE, these therapies are not approved for MPE, and their primary prescription remains in the subset of patients with intermediate high-risk, intermediate low-risk, and SMPE. The FlowTriever All-Comer Registry for Patient Safety and Hemodynamics (FLASH) revealed that many patients who were identified to have normotensive cardiogenic shock did not progress to fulminant cardiac failure, indicating that perhaps early catheter-directed thrombectomy may slow the evolution to MPE.^13,19^

## VA-ECMO

For MPE, one of the most promising therapies in terms of survival is VA-ECMO. MPE involves obstructive shock from a thrombus as well as the presence of RV failure. VA-ECMO provides cardiac support to reduce RV preload and shunt blood directly into the ECMO pump, and then into the iliac artery, to provide retrograde cardiac support. This allows hemodynamic optimization of the patient with subsequent removal of the device. If the device cannot be separated, advanced therapies such as catheter-directed thrombolysis and thrombectomy, as well as surgical pulmonary embolectomy while on VA-ECMO support, can be provided to facilitate separation.

In appropriately triaged patients who received VA-ECMO up front, Pasrija and colleagues demonstrated remarkable survival of 95% at 1 year, with 1 death out of 20 patients.^32,33^ Similarly, in a case series evaluating trends for VA-ECMO use in patients with MPE, VA-ECMO was shown to be independently associated with improved mortality.^33^ The continued case series, reporting improvement in outcomes, led to a discussion at the 2018 American Thoracic Society meeting recommending that patients with MPE be transferred to regional centers for evaluation for ECMO.^34,35^ As PERTs have become more present in the triage and treatment of MPE, the incorporation of VA-ECMO into the treatment algorithm has improved the safety and outcomes for MPE patients.

## Conclusion

In summary, MPE remains a difficult clinical entity to recognize, triage, and stratify. The current definitions of MPE may be causing patient harm as they may lead to many patients missing guideline-directed thrombolysis. The earlier MPE is identified, the lower the complication rate and the higher the chance of survival. MPE can be risk stratified with the addition of plasma lactate into the risk scoring systems (sPESI, PESI, BOVA, and TELSO) to improve recognition of early mortality risk in high-risk PE patients.

Much work remains to be done in improving the management and outcomes of patients with MPE. It starts with reaching a consensus on criteria for a safer approach to improve clinical recognition of MPE and a structured decision-making algorithm.

## Key Points

- The American Heart Association and European Society of Cardiology have different classification schemes for pulmonary embolism (PE).
- Misdiagnosis of PE is common, occurring in 30% of emergency departments initially and in up to 40% of intensive care unit patients, leading to potentially fatal consequences.
- Various scoring systems, including Pulmonary Embolism Severity Index (PESI), Simplified PESI (sPESI), BOVA score, TELOS score, and SHIELD score help predict mortality and guide treatment decisions for different subtypes of PE.
- The definition of massive PE (MPE) deserves reconsideration to trigger a more rapid recognition and assessment of the severity of a patient’s clinical symptoms.
- Treatment for MPE typically involves a combination of anticoagulation, thrombolysis, and in severe cases, surgical pulmonary embolectomy with consideration given to advanced therapies such as catheter-directed interventions and venoarterial extracorporeal membrane oxygenation.
- Despite the potential benefits of thrombolysis and other advanced therapies, there is underutilization, with many MPE patients not receiving these treatments, emphasizing the importance of pulmonary embolism response teams in optimizing care.
